# Development and feasibility of a home-based education model for families of children with sickle cell disease

**DOI:** 10.1186/1471-2458-14-116

**Published:** 2014-02-05

**Authors:** Catherine Hoyt Drazen, Regina Abel, Terianne Lindsey, Allison A King

**Affiliations:** 1 Washington University in St. Louis School of Medicine, Program in Occupational Therapy, 4444 Forest Park Pkwy, Box 8505, St. Louis, MO 63108, USA; 2 St. Louis Children’s Hospital, One Children’s Place, St. Louis, MO 63110, USA; 3 Department of Pediatrics, Division of Hematology and Oncology, Washington University School of Medicine, Campus Box 8505 4444 Forest Park Ave., St. Louis, MO 63108-2292, USA

**Keywords:** Sickle cell disease, Development, Early intervention, Parent education, House calls

## Abstract

**Background:**

Children with sickle cell disease (SCD) commonly have cognitive deficits, even among toddlers. Much medical literature emphasizes disease-based factors to account for these deficits. However, the social environment plays a large role in child development. To address the specific needs of early childhood, a monthly hospital-based education program was initiated to educate parents about child development. Education sessions were poorly attended (20-25%) and deemed unsuccessful. This study describes the development and implementation of a home-based education service to teach parents about SCD, developmental milestones and positive parenting techniques.

**Methods:**

This was a prospective, single-arm intervention to study the feasibility of a home-based caregiver education program for families with infants and toddlers with SCD. Parents of children aged 0-3 years with SCD from one Midwestern hospital were approached to participate in a home-based program. The program followed the Born to Learn™ curriculum provided through the Parents as Teachers™ National Center. Reminder calls or texts were provided the day before each visit. Results of the first twenty-six months of the program are presented.

**Results:**

A total of 62% (56 of 91) of families approached agreed to participate; all were African American. The majority of caregivers were single mothers with a high school education or less and whose children had Medicaid for health coverage. The phenotypes of SCD represented in this sample were similar to those in the general SCD population. Over 26 months, 39 families received at least one home visit. Parents of infants (younger than 8 months) were more likely to participate in the home-based education program than parents of older children, (Fisher’s exact test, p < .001).

**Conclusions:**

For participating families, home-based visits were a feasible method for reinforcing clinic education. About 43% of eligible families participated in the education, a two-fold increase in the poor attendance (20%) for a previous hospital-based program. A home visitation program for parents of infants with SCD could offer an effective approach to helping these children overcome adverse environmental conditions that are compounded by the complexities of a chronic health condition.

## Background

In the United States (US), approximately 100,000 people live with sickle cell disease (SCD). The majority are African American [1]. SCD is an inherited blood disorder that causes red blood cells to be brittle, sticky and crescent shaped. Sickled cells have a shorter life span than normal red blood cells, and affected persons have chronic anemia. The abnormal cells are more likely to become trapped in blood vessels, causing vaso-occlusion and pain, the most common morbidity associated with the disease [2]. Other complications include cerebrovascular disease (stroke and cerebral infarcts), splenic sequestration (blood pools in the spleen), dactlyitis (swelling of the hands and feet), priapism (prolonged erection), acute chest syndrome and necrosis of the hip [3,4].

There are several forms of SCD that vary in prognosis and severity; the most prevalent and severe is hemoglobin SS (HbSS). In the US, an estimated 1 in 500 African American live births have the disease [1]. Additionally, approximately 1 out of 12 African Americans carry S trait. Therefore, SCD is one of the most common genetic disorders affecting people in the US, with approximately 3.4 million carrying the trait.

SCD is associated with an increased risk for cognitive deficits that can impact academic performance [5]. Compared to children with normal hemoglobin, children with SCD are far more likely to have a cerebrovascular accident (CVA) [6]. Approximately 40% of children with HbSS will have a silent cerebral infarct [7,8] or an overt stroke by adulthood [6,7,9]. Compared to children with no brain abnormalities (as confirmed by MRI examination), children with a history of CVA have significantly lower full scale intelligence quotient (IQ), verbal IQ, performance IQ and math achievement [10]. Over half of children who have had a silent infarct will require special services in school or be retained a grade level, indicating poor academic achievement and more subtle cognitive impairment [11]. However, developmental delay cannot be attributed solely to CVAs. Full scale IQ testing has reported that children with SCD and no MRI abnormalities have an IQ between 85 and 90 [10]. Furthermore, over a quarter of children with SCD and no cerebral insult required special services at school or needed to repeat a grade [11,12].

Developmental delay for children with SCD has been observed as young as nine months of age [13,14]. By 24 months, nearly 40% of children with SCD are deemed to be at risk for clinically significant developmental delay [15]. By three to four years of age, up to 50% of children with SCD have delays [16]. Although developmental delay in children with SCD has been documented in several studies, the cause of delay is not clear. SCD alone does not account for poor academic outcomes [17]. Disease severity and environmental risk factors combine to influence the outcomes of children with SCD. A recent model of school-aged children with SCD showed that the educational status of a parent actually contributed more to a child’s full scale IQ than the presence of a silent cerebral infarct [18].

Children with SCD face more environmental challenges than most. Many children who suffer the physical effects of SCD also live in dangerous, impoverished neighborhoods and have limited access to educational opportunities [19]. Children living in poverty are at an increased risk for deficits in cognition, language and school readiness [17,20]. By three years of age, children growing up in low-income households have smaller vocabularies than their more advantaged peers [21]. Language delays severely impact children’s ability to participate in school and as a result, children in poverty have lower academic achievement [20]. Children growing up in poverty often have limited exposure to materials, experiences, and environments that can influence the achievement of developmental milestones and have a significant positive impact on school readiness [22-25]. The quality of the home environment, including parenting techniques, has been shown to mediate the influence of the neighborhood and the child’s cognitive abilities as early as age three [26,27].

### Previous interventions

The local SCD program receives an average of 25-30 newborns each year. We initiated a monthly, Saturday morning hospital-based parent education program to address educational needs of families that were new to the clinic. Families with children under 36 months of age were invited to attend at clinic visits, mailed letters and called to confirm attendance if they had indicated interest. The sessions were held if there was a minimum of three confirmed attendees. The total number of children (newborn to three years) for that period was 100-120. Over a period of 21 months, 25 families attended one education session. Thus, only 20-25% of the families of children in that age group received one educational session. However, nine sessions had no attendees and half had only one family despite reminder phone calls with confirmed attendance. The low rate of attendance demonstrated that the hospital-based, Saturday parent education and developmental screening was not feasible for this population.

### Current intervention

Prior to the present intervention, few of the young children with SCD treated at our SCD clinic were receiving early intervention or parent education services such Parents as Teachers™, despite eligibility. Parents as Teachers™ is a home-based parent education curriculum that aims to provide information, support and encouragement to help children reach developmental milestones during the first few years of life. Parents of children with SCD in our center were unaware of available resources and were exposed to a high number of daily stressors including poverty, highly mobile households, overly crowded homes and community violence. Among pre-school-aged children with SCD, psychosocial factors may have a greater impact on early childhood development than sickle cell disease related factors [16]. In order to ameliorate these challenges among the families of infant/toddlers with SCD, we proposed a home-based parent education program to reinforce information regarding SCD provided in the clinic as well as address developmental milestones.

We implemented a home-based education model that might eliminate many of the barriers to participation in a hospital-based educational program for parents of children with SCD. A home visitation model would enable the clinic team to better determine factors related to the home environments that could affect development and the ability of the caregivers to respond to the needs of their children with SCD. The purpose of the current study was to determine if a home based parent education program targeting parenting skills and typical developmental milestones was feasible as defined by 50% consent rate for those recruited for the study and at least 50% completion of scheduled home visits.

## Methods

The current study was a prospective, single arm intervention. Approval was obtained from the Institutional Review Board of Washington University School of Medicine. Participants were recruited from the local SCD program. At our clinic, newborns are initially seen at about two months of age and return appointments are approximately three months apart. Older children may be seen every four to six months.

### Participants

#### Inclusion criteria

All participants had a confirmed diagnosis of SCD and were active patients at the clinic. Children were between the ages of 3-36 months at the time of recruitment, lived within 30 miles of the hospital and caregivers spoke English fluently. The parent/primary caregiver provided consent for participation.

#### Exclusion criteria

Patient/caregiver dyads were excluded if the primary caregiver did not have stable housing.

### Recruitment

Caregivers of all eligible children were approached during regularly scheduled visits to the clinic. Families of newborns were approached for the current study after their second or third clinic visit, typically when the child was between four to six months of age. Older children and their caregivers were approached at their first visit following the initiation of the study. Caregivers were offered the opportunity to participate in an accredited Parents as Teachers™ (PAT) Born to Learn curriculum provided by an occupational therapist that was certified as a PAT provider and was educated about risks associated with SCD.

### Retention

Upon consent, a date was scheduled for the educator to visit the family’s home. Families received reminder phone calls the day before their scheduled visit and visits were rescheduled as needed. During home visits, the educator addressed caregiver concerns regarding SCD and development. Caregiver education focused on developmental milestones and age appropriate skill-learning activities during infancy and toddlerhood that might mediate some of these effects.

Caregivers were encouraged to participate in play and reading to their child during the visit and were asked to bring up any concerns. Most visits lasted approximately one hour. Every visit incorporated an age-specific activity to challenge emerging skills, handouts about development and a book for the child to keep. Books were donated to the program.

### Tools

#### Parents as Teachers™ Born to Learn

Parents as Teachers (PAT) is an internationally recognized educational curriculum for children 0-36 months and their caregivers that was developed to teach parents skills to help them engage with their child and increase awareness of developmental milestones (http://www.parentsasteachers.org). The PAT program has previously been shown to increase school readiness [28]. PAT utilizes a home-based visitation method in which a trained parent educator goes to the home at least once a month. The curriculum provides activities and handouts based on the child’s age. The parent educator addresses topics relevant to development at the child’s specific age and discusses emerging skills for the parent and child to work on in the coming weeks. The parent educator also assists families in getting connected with local community organizations and available resources.

#### Educational materials

The parent educator selected additional handouts as appropriate for each family’s needs. Families reviewed SCD information through handouts, flipcharts and videos. Handouts were created by the team to help families understand how to manage physical activities, changing seasons and cold weather with a child with SCD. Additional support materials were used as needed such as the Act Early program provided from the Centers for Disease Control and Prevention (CDC) [29]. The CDC provides informational brochures, handouts and books about developmental milestones that are available at no cost through their website.

#### Outcome measures

Demographic information was collected from the primary caregiver and medical records upon enrollment in the current study. Feasibility was determined by the acceptance (families that were approached for participation compared to the number that consented) and the number who actually participated in a home visit. The number of scheduled visits completed was also recorded. Participating families were asked to complete a satisfaction survey after completing a minimum of four home visits. Field notes were taken following each home visit. Notes included documentation of the handouts that were provided, who participated in the visit, topics discussed and the child’s current level of functioning in intellectual, language, motor and social-emotional development.

## Results

All families were African American. As shown in Table 1, the majority of families were living at or near poverty as indicated by the percent (82%) that received health care coverage via Medicaid. One fifth of families who participated had three or more children under the age of five years living in the home.

**Table 1:** Demographics of families that completed a visit as of 12/31/2012 (N = 39)

**Variable**	
Age of child in months at consent (Mean)	9.2 (range: 2-35 months)
Participation rate of families with children at age 7 months or less	20 (87%)
Participation rate of families of children at age 8-36 months	19 (58%)
Gender (male)*	21(54%)
Phenotype of child	
HbSS	19 (49%)
HbSC	16 (41%)
Other (Persistent fetal hemoglobin, beta-thalassemia)	4 (10%)
Medicaid health care coverage for child	32 (82%)
Marital status of parents: unmarried	34 (87%)
Average age of primary caregiver at enrollment in years	27 (range:15-49)
3 or more children under 5 years in household	8 (20.5%)
Primary caregiver education	
Less than high school graduation	8 (21%)
High school diploma or GED	15 (38%)
Some college	10 (26%)
College graduate	6 (15%)

### Consented vs. Non-consented families

There was no significant difference in sickle cell phenotype between those who participated in PAT and those who chose not to participate, (Hb SS, 50% vs. 58%; Mann-Whitney U, p > .2). There was also no significant difference in the insurance coverage between those who participated in PAT and those who did not, (Medicaid, 77% vs. 71%; Mann-Whitney U, p > .9). Similar distribution of SCD phenotype and economic status (as measured by insurance provider) indicate that non-participants did not vary significantly from families who participated.

### Parents of younger children were more likely to schedule a home visit

All children who met inclusion criteria were approached (N = 91). Over a period of 26 months, 56 families with a total of 58 children (64% of those eligible) consented to participate. Of those 58 children, a visit was scheduled for 39 (70%). Table 2 indicates that significantly more families consented if children were 2-7 months of age than if children were 8-36 months of age (77% vs., 62%, respectively, Fisher’s exact test p < 0.05). For those who consented, significantly more visits were scheduled if the child was seven months of age or younger than if the child was more than seven months of age, (87% vs. 58%, respectively; Fisher’s exact test p < 0.001).

**Table 2:** Number of families that scheduled home visits based on age of child at time of recruitment

	**At least 1 PAT visit N**	**No PAT visits N**	**Did not consent N**
Children 2-7 months	20	3	7
Children 8-36 months	19	14	28

Thirty-nine families participated in at least one home visit. Sixteen families (41%) had between 1-5 visits, thirteen (33%) had between 6-12 and ten (26%) families had over 13 visits to the home. Over this time, nine children aged out of the program, three parents scheduled in person but never answered the phone to confirm, and two have been lost to follow up because they moved. Of those that completed a visit, at least 50% depended on other forms of state or government assistance such as a supplemental nutrition program, food stamps or Social Security Income. For families that were lost, the cause was most often that the phone number had changed and the family could not be contacted. A social worker was contacted to help locate families for medical care. Over the past 26 months, 15-24 families actively participated each month.

The age of children of families that did not consent was obtained through retrospective analysis of the patients’ appointment records. When the program was initiated, families of older children were called because clinic visits are less frequent.

#### Evaluation of PAT program

Participating families were asked to complete a satisfaction survey of the home visitation program after participating in the program for at least four visits. The parent educator assured them that evaluations were anonymous and they could mail them in or give them to the nurse practitioner in the clinic. In one circumstance, the parent struggled with low literacy and the parent educator offered to read the statements aloud and write in answers for them. Caregivers were asked to check the box that describes how they feel on a Likert scale of one to five ranging from strongly agree to strongly disagree. Of the 23 families who completed more than four visits, 13 evaluated the program. All reported that they agree or strongly agree that they like PAT visits and that they strongly agree that PAT visits helped the caregiver understand development and engage with their child. There were two open-ended questions asking what aspect of PAT they liked best and if they could make changes, what would they be. No one recommended changes.

### Qualitative answers to evaluation

One parent of a 20 month old stated in her evaluation “I read to her because you kept telling me to. And you know, she brings me books. She likes it”. When this child was 8 months old the mom was initially hesitant to read to her infant because she did not like to read and she did not believe that her daughter would enjoy it. Another parent stated, “I like having the visits. She (parent educator) gives me ideas how to play with my child”. One mom of a 10 month old said “I feel better now that I understand more about SCD. I’m not as scared anymore”.

### Recruitment and program retention

Recruitment was continuous throughout the study period; therefore the number of visits per family is not reflective of the number of families that are currently active in the program. For the 36% of families that elected not to participate in this free program, most stated that they did not feel that they had time, did not have consistent housing, or did not feel that they needed the services. During the study period, nine children aged out of the program (> 36 months of age) and could no longer receive visits. Additionally, four families requested to stop services, and three were lost to follow up.

**Figure 1:**
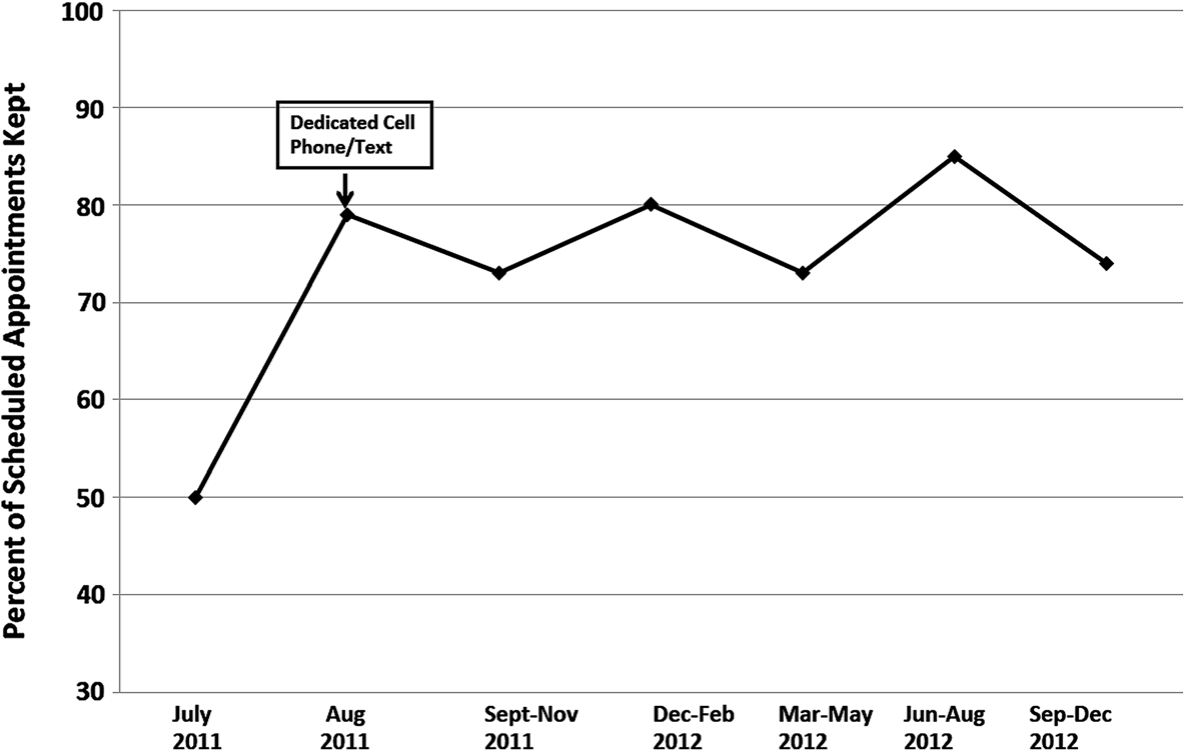
Percentage of scheduled home visits completed.

The most common barrier was maintaining contact with families. When the family could not be reached to confirm, visits were not completed. Visits were rescheduled often; the most common reasons were that the child was hospitalized or a change in the caregivers’ schedule. During the first six months of the program, only about 50% of scheduled visits were completed. Initially, all calls were made from an office phone affiliated with the hospital or university. Beginning in the seventh month of the program, we incorporated a dedicated cell phone to contact families. In the one-month period prior to acquiring the cell phone, 9 of 18 scheduled visits were completed (50%). That rate was representative of the number of scheduled visits completed when using the university-based landline. A cell phone was obtained under the name “Sickle Cell” with texting capabilities in August 2011. Rate of adherence to scheduled sessions increased from 50% to 79% after inclusion of the cell phone to contact families prior to the home visits (Figure 1). Adherence remained at 77.3% for the remainder of the study (months 8-24).

A cell phone was obtained under the name “Sickle Cell” with texting capabilities in August 2011. Rate of adherence to scheduled sessions increased from 50% to 79% after inclusion of the cell phone to contact families prior to the home visits. Adherence remained at 77.3% for the remainder of the study (months 8-24).

### Home visits

Qualitative observance of parenting practices revealed at least three common needs across many of the families, including lack of appropriate toys, failure to read/talk to the child, and inability to deal with challenging child behaviors during mealtime and bedtime. During home visits, strategies were discussed with caregivers about how they could engage with their child using pictures, books, or common items around the home. Table 3 lists some of the outcomes observed from these discussions. Examples of ways to play with items around the home, such as coffee cans, juice bottles or paper plates were demonstrated. Parents also had opportunities at each visit to discuss concerns they might have and referrals were made to community resources to address any urgent needs the family may have such as food, birth control, health care, lead testing, and employment. These discussions helped build rapport and trust between the provider and the family.

**Table 3:** Barriers to developmental progress in young children with SCD and interventions

**Challenge**	**Intervention**	**Result**
Lack of developmentally appropriate toys.	Handouts with pictures of appropriate toys for age. Discussion about developmental milestones and purpose of play.	Minimum of 8 families made toy purchases based on recommendations.
Reading/talking not incorporated into routine.	Provide minimum of 1 book per visit. Discuss value of reading and demonstrate reading to a child. Emphasize importance of looking at books even to just talk about pictures. Make homemade books with Zip top sandwich baggies and pictures.	Minimum of 6 children have books incorporated into daily routine.
Challenging child behaviors.	Discussion about typical behaviors and strategies on how to manage them. Discussion of how to implement routines.	Minimum of 4 children have established a routine in their day.

### Home visits and relation to sickle cell education

The parent educator was trained and educated on the genetic inheritance of SCD, morbidities associated with the disease and their impact on child development. The parent educator had the hospital version of the parent education program available with her at all times to review if families expressed need. The parent educator was able to reinforce training provided during visits to the sickle cell clinic such as how to palpate for an enlarged spleen, what temperature to monitor for and how to identify dactylitis. Several caregivers had questions regarding medications such as penicillin and folic acid and what they were for. Parents were directed to call the SCD clinic with any medical questions or concerns.

## Discussion

This study provides preliminary data indicating that a home-based program can be a feasible method for education of parents of infants with SCD. Given the prevalence of SCD and the risks for significant delay, a reliable method for providing early intervention to families of children with SCD is greatly needed [13,15,30-34]. Providing education at the hospital regarding parenting techniques and developmental milestones was previously not successful because of barriers concerning transportation and work schedules. A home-based program to provide services to these families may be more successful and improve outcomes for these children.

Recruitment and retention were primary concerns when initiating this pilot program. Since enrollment was continuous, families initiated visits at different times and consequently have varying numbers of visits to date. Parents of younger infants were more likely to commit to the parenting program. Possibly, these parents are more open to suggestions and education because they are eager to maximize their child’s health and development in the face of a newly diagnosed chronic disease. Initially, visits were scheduled with families in advance and the parent educator went to the home at the scheduled time. Unfortunately, there was a high incidence of uncompleted visits due to families not being home or forgetting their scheduled appointment. Reminder phone calls the day prior to a visit increased the completion rate substantially, but there was still significant difficulty communicating with some families, particularly younger parents. Consequently, text message reminders were implemented for parents that indicated that texting was a convenient form of communication. Using a combination of reminder phone calls and texting greatly improved retention, particularly for younger caregivers who preferred texting to phone calls or had unlimited texting plans but minimal or no minutes available for phone calls. With this system, the parent educator did not go to the home unless a family confirmed the visit and services were terminated if a family was not home for three scheduled and confirmed visits.

While several studies have documented the developmental delay of young children with SCD, few, if any, interventions have been documented to ameliorate these challenges. Home based interventions enable providers to connect with caregivers and identify aspects of their environment that can be used for learning and describe these benefits individually for the child within their natural environment. A formal parenting program fills a gap in our current education plan for the parents of children with SCD, addressing both the medical and psychosocial needs of the children. Most of the families that agreed to participate in the program scheduled and completed multiple visits, and many of them remained active in the program.

In our observation, families of children with SCD often struggle with many challenges that they do not identify or reveal within a clinic visit. We observed that many caregivers have not had the opportunity to learn parenting strategies and they appreciate the information, encouragement and praise for their actions such as providing support and encouragement when family members stop smoking in the home or acknowledging family members engaging the child in conversation or interactive play. Further, caregivers seemed to appreciate having their challenges recognized and being given tools to advocate for themselves and their children. It is of utmost importance that providers are trained in cultural sensitivity and communication to adequately meet these families’ needs.

Caregivers verbalized that they did not understand the purpose of medications or various treatments, and many admitted to not being adherent to suggestions. The Health Belief Model describes the importance of considering one’s understanding of a health related issue and adherence with medical advice [35]. This model applies to our population and helps to explain caregiver insecurities or disinterest in a parent education program. Possibly, many parents do want the best for their child, but do not perceive that there is serious risk for their child, or they may not understand that the child may have challenges that are necessary to address. Additionally, caregivers may not fully trust people affiliated with the medical community. Lack of understanding, perception of risk or distrust may affect caregivers’ willingness to communicate and participate in a parent education program.

The cost of this program included the salary of the primary provider, which in this case was an occupational therapist. It would be possible for future programs to use alternative providers such as child life specialists, social workers, or those with qualified training in child development and SCD. Associated costs to the implementation of this program included mileage for the provider, materials for home visits and training in the PAT™ curriculum. Additionally, in this sample we identified that families of newborns were more likely to be active participants in this program and it is possible that a more targeted program could be more cost effective. Future directions can include evaluation of the impact of the program on child development, parental knowledge of SCD and health care utilization.

### Limitations

This pilot study had several limitations. As a single center, single arm intervention, generalizability is limited. However, for our purpose, we learned that families are interested in early childhood and parenting and are willing to welcome an educator into their homes. The satisfaction surveys were given to families following a home visit, which may have biased caregivers to answer more positively since many completed them immediately. Families were encouraged to keep evaluations anonymous and fold them up when they were completed. Another limitation of this program was that it was not coordinated with the school system. We chose to have a private PAT provider to ensure that each family would be able to receive services regardless of school district staffing or budget restrictions. This method was effective in providing services but required more time to help families get involved with other community organizations. Caregivers who choose not to participate in home-based parenting interventions can be provided information about local community or online resources for education and support. Despite limitations, this pilot study demonstrated that in our location, families are interested in participating in a home-based parent education program.

## Conclusions

Children with SCD are a vulnerable population. With a home-based program, we were not only able to achieve a two-fold increase in a single SCD education session but were also able to provide a monthly intervention. The ongoing visits facilitated the development of a trusting relationship that permitted the parent educator to identify barriers to developmental progress previously unrecognized in the clinic. Based on observations and discussions with parents during the study, many of the families who care for a child with SCD struggle with understanding typical developmental milestones and lack knowledge of activities that encourage and challenge the child to meet these goals. Home-based services that address parenting skills and therapeutic activity along with repetition of concerns specific for SCD are a feasible way to reach this population. A dedicated cellular phone increased retention by providing reminder phone calls and text messages. The convenient communication opportunities from text messaging were well received. Providing skilled educational and supportive services in the home is also beneficial by helping parents make modifications to the home environment to increase safety and accessibility to appropriate activities by the child. More research should be conducted to determine the effects and outcomes of children receiving this intervention. A home evaluation of parent interaction, environment, and child development at baseline and following the intervention would objectively demonstrate the outcomes of providing in home services to this population.

## Abbreviations

SCD: Sickle cell disease, including children with sickle cell anemia and other hemoglobin variations including HbSS, HbSC, Hb beta thal; PAT: Parents as Teachers®. A home visitation program and curriculum for children 0-3 years.

## Competing interests

The Health Resources and Services Administration (U1EMC17182) funded this study. This publication was made possible by Grant Number UL1 RR024992 from the National Center for Research Resources (NCRR), a component of the National Institutes of Health (NIH) and NIH Roadmap for Medical Research. Its contents are solely the responsibility of the authors and do not necessarily represent the official view of NCRR or NIH.

## Authors’ contributions

CH designed the study, served as the parent educator, and drafted the manuscript. RA designed the study, analyzed results, edited the manuscript and supervised CH. TL assisted with participant recruitment, educated parents and edited the manuscript. AK conceived of the study, participated in the design and coordination, analyzed the results and edited the manuscript. All authors read and approved the final manuscript.

## Authors’ information

**Catherine Hoyt:** Catherine Hoyt OTD, OTR/L is an occupational therapist and works in the Child Health and Education Laboratory in the Program in Occupational Therapy at Washington University School of Medicine and was the parent educator/home visitor for this project.

**Regina Abel:** Regina Abel PhD has a doctoral degree in Developmental Psychology and was the supervisor for this program. Dr. Abel works in the Child Health and Education Laboratory in the Program in Occupational Therapy at Washington University School of Medicine.

**Terianne Lindsey:** Terianne Lindsey CPNP, RN was the nurse practitioner for the newborns and toddlers with SCD.

**Allison King:** Allison King MD, MPH is the PI of the Child Health and Education Laboratory and is principal investigator of this project through the Program of Occupational Therapy at Washington University School of Medicine. She is also a pediatric hematology/oncology physician.

## Pre-publication history

The pre-publication history for this paper can be accessed here:

http://www.biomedcentral.com/1471-2458/14/116/prepub
